# Acute Respiratory Distress Syndrome Following Minimally Invasive Cardiac Surgery

**DOI:** 10.7759/cureus.78793

**Published:** 2025-02-09

**Authors:** Takashi Nagase, Noriyuki Kashiyama, Masahiro Ryugo, Osamu Monta, Shinichiro Oda

**Affiliations:** 1 Cardiovascular Surgery, Fukui Cardiovascular Center, Fukui, JPN; 2 Cardiovascular Surgery, Kyoto Prefectural University of Medicine, Kyoto, JPN

**Keywords:** acute respiratory distress syndrome [ards], cardiovascular and thoracic surgery, minimally invasive surgeries, perioperative cardiovascular evaluation and management, respiratory support

## Abstract

Acute respiratory distress syndrome (ARDS) incidence following minimally invasive cardiac surgery (MICS) is rare. We report a case of acute respiratory failure following cardiac surgery that was diagnosed as ARDS. A 77-year-old female patient diagnosed with aortic valve stenosis underwent aortic valve replacement via a right thoracotomy. The surgery was uneventful, and the patient was extubated on the day of surgery. However, oxygen saturation steadily declined one day postoperatively, and the patient was re-intubated. Chest radiography revealed bilateral heterogeneous infiltrates. After excluding other differential diagnoses, we diagnosed ARDS based on established diagnostic criteria. Inhalation of nitric oxide, methylprednisolone, and Sivelestat were initiated. The patient’s respiratory status gradually improved, and she was re-extubated eight days postoperatively. She remained stable in the general ward and was transferred for rehabilitation. Early diagnosis and intervention are key for favorable outcomes in these cases.

## Introduction

Acute respiratory failure is a severe complication of cardiac surgery, and severe lung injury following cardiopulmonary bypass remains a significant cause of morbidity and mortality, with a substantial impact on healthcare expenditures [1,2]. However, accurately diagnosing the underlying cause of respiratory failure can sometimes be challenging, which may lead to mismanagement of treatment strategy. In this report, we present a case of respiratory failure following minimally invasive cardiac surgery (MICS) that was ultimately diagnosed as acute respiratory distress syndrome (ARDS).

## Case presentation

**Figure 1:**
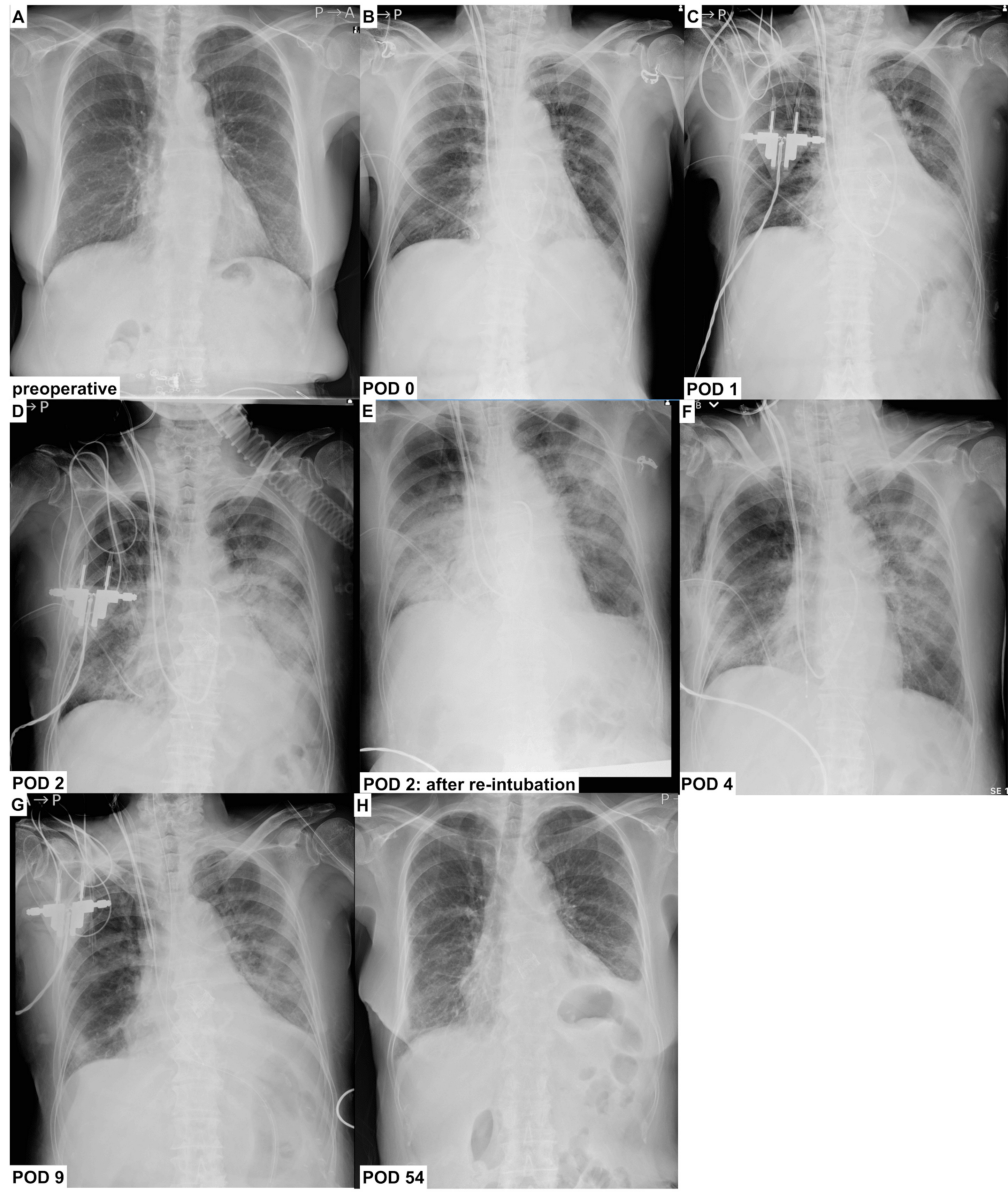
Changes in chest radiography through disease course A: preoperative X-ray; B: POD 0; C: POD 1; D: POD 2; E: POD 2, after re-intubation; F: POD 4; G: POD 9; H: POD 54 POD: postoperative day

A 77-year-old female patient diagnosed with severe aortic stenosis was referred to our hospital for cardiac surgery. Her medical history included diabetes mellitus and she was not a smoker. The ejection fraction was found to be 64%. Preoperative spirometry results were within normal ranges. Figure 1A shows the preoperative chest radiograph.

Right thoracotomy was performed through the fourth intercostal space to access the pericardial cavity. Cardiopulmonary bypass (CPB) was performed via the right femoral artery and veins. After aortic cross-clamping, the bioprosthetic valve was selected and implanted. CPB weaning was uneventful. The CPB and aortic cross-clamp times were 120 and 76 min, respectively. Transfusions were performed during surgery. A Swan-Ganz catheter was routinely inserted, and the cardiac index (CI) and mixed venous oxygen saturation (ScvO_2_) were monitored continuously. Figure 1B shows a chest radiograph obtained on postoperative day (POD) 0.

The patient’s respiratory and circulatory statuses remained stable, and she was extubated on the day of surgery. However, oxygen saturation steadily declined one day postoperatively. Maintaining oxygen saturation under bi-level-positive airway pressure was difficult, and she had to be re-intubated after POD 2. Positive end-expiratory pressure was set at 10 cmH_2_O during mechanical ventilation. The P/F ratio at the time of re-intubation was 61 mmHg. Chest radiography revealed bilateral and heterogeneous infiltrates (Figures 1D-1F).

**Figure 2:**
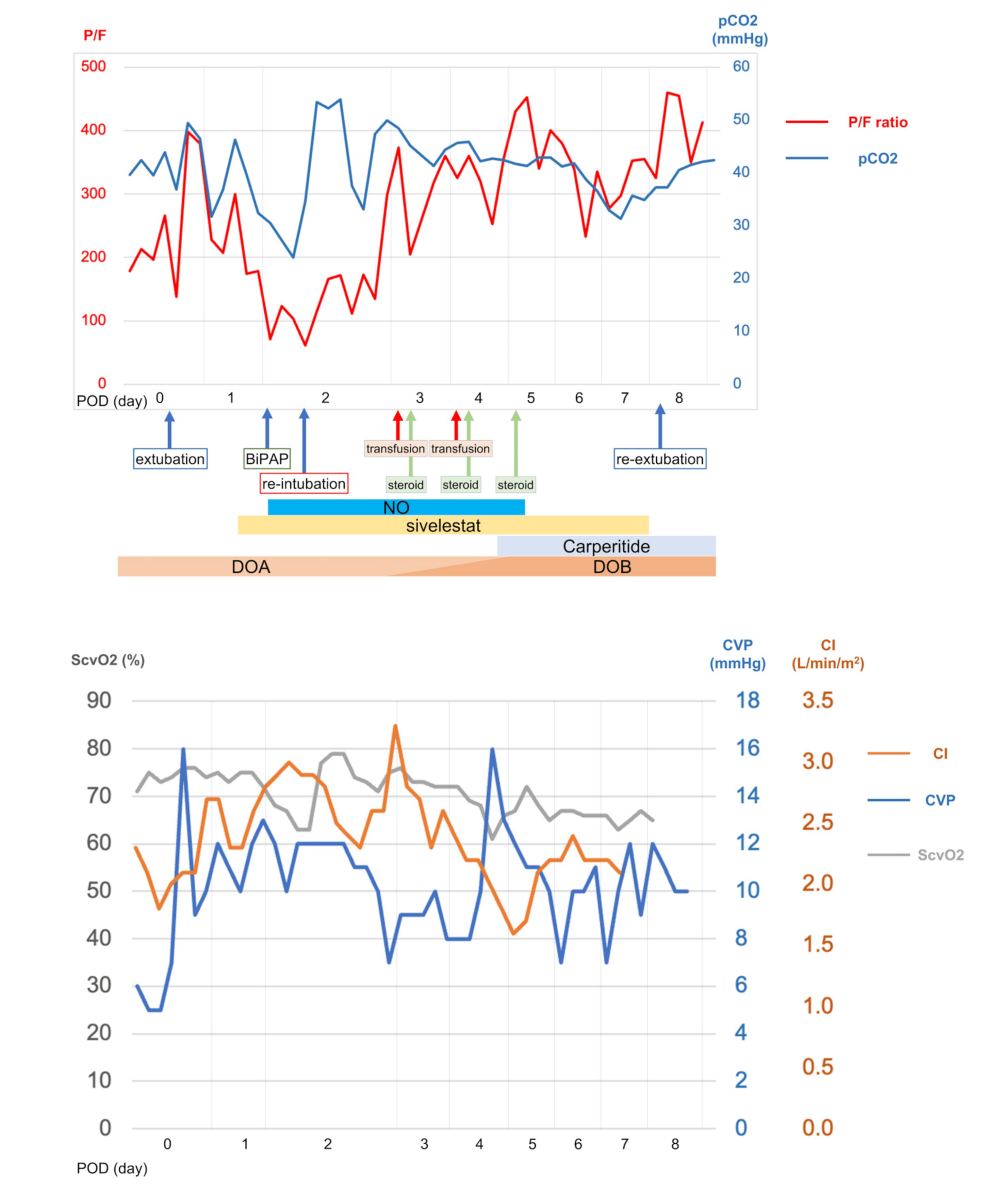
Summary of the evolution of patient’s vitals and data of arterial blood gas analysis through disease course POD: postoperative day; BiPAP: bi-level positive airway pressure; NO: nitric oxide; DOA: dopamine; DOB: dobutamine; CI: cardiac index; ScvO_2_: mixed venous oxygen saturation; CVP: central venous pressure

**Figure 3:**
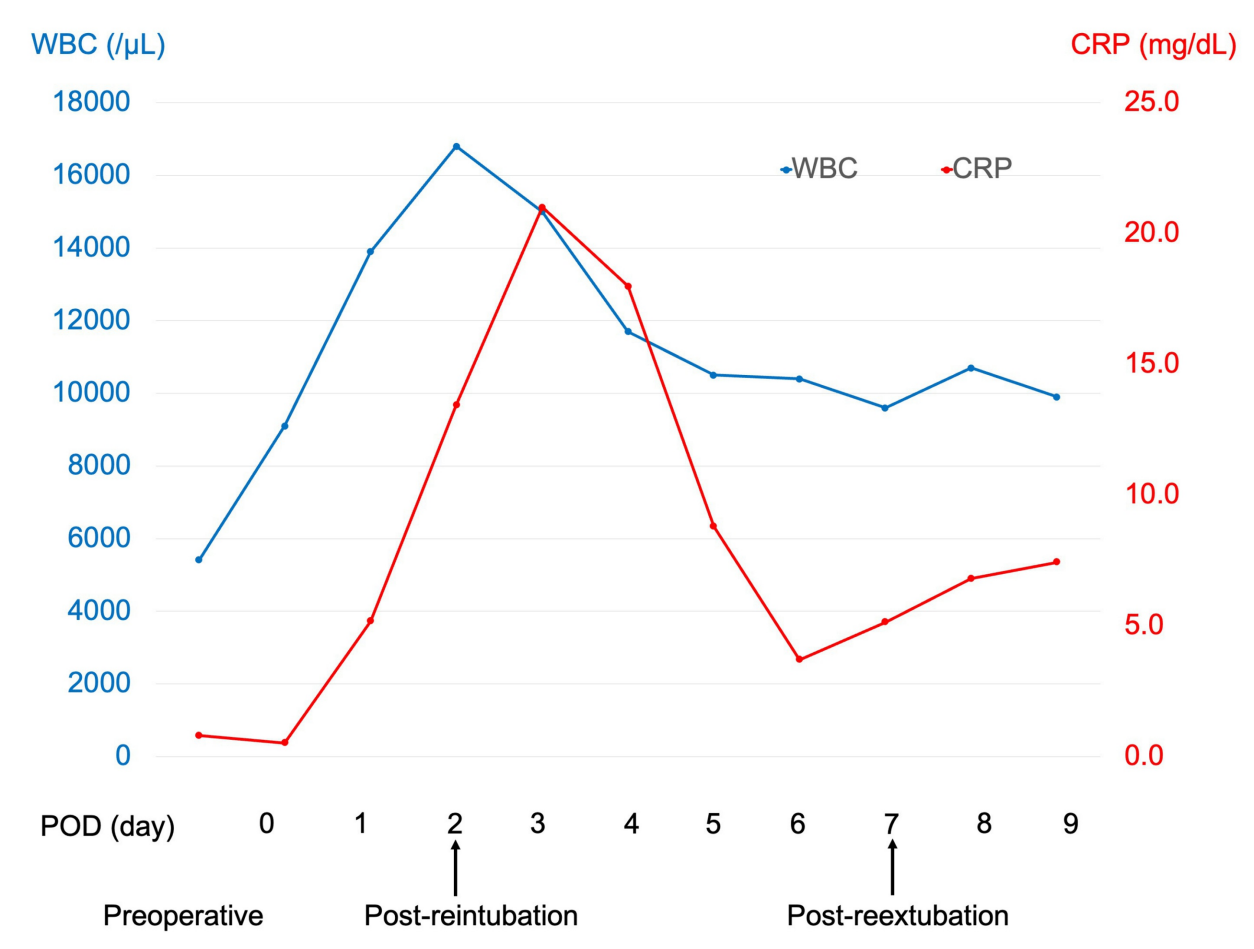
Time course of postoperative inflammatory response WBC: white blood cells; CRP: C-reactive protein; POD: postoperative day

We suspected bacterial pneumonia, and subsequently, for the treatment of bacterial pneumonia, the antibiotic regimen was escalated to tazobactam/piperacillin from cefazolin at POD 5. At the same time, the sputum culture examination revealed no bacterial growth. Inhalation of nitric oxide (NO) and Sivelestat were administered. Methylprednisolone was administered as follows: 1000 mg on POD 2, 40 mg on POD 3, and 125 mg on POD 4 and POD 5. Bedside echocardiography revealed normal left heart motion with no evidence of prosthetic valve failure. CI, ScvO_2_, and central venous pressure (CVP) were within normal ranges. After re-intubation, oxygen saturation steadily improved with the weaning off of ventilation pressure. Sivelestat infusion was terminated on POD 7, and the patient was extubated on POD 8. Figures 2-3 depict a summary of the evolution of the patient’s vitals, arterial blood gas data, and inflammatory data through the disease course. She remained stable in the general ward and was transferred for rehabilitation after POD 78.

## Discussion

Acute respiratory failure after MICS is a rare but lethal condition. However, diagnosing the cause of respiratory failure following MICS is sometimes difficult. Acute bacterial pneumonia is one of the major complications following cardiac surgery. In particular, ventricular-associated pneumonia (VAP) is the most commonly observed infectious comorbidity in the ICU [2]. Initially, we continued treatment with a presumption of bacterial pneumonia. However, based on the poor response to antibiotics and sputum culture results, we retrospectively concluded that the likelihood of bacterial pneumonia was low. In addition, early extubation within 24 hours from the surgery was achieved in this case, which did not align with the definition of VAP [3].

Cardiogenic pulmonary edema (CPE) is a major complication of cardiac surgery. Patients in the perioperative course are prone to experiencing CPE because of low cardiac output after cardiac arrest and the influence of CPB or heart failure. Therefore, proper fluid management and the use of adequate inotropic agents to optimize oxygen delivery are crucial [4]. In this case, the postoperative hemodynamic parameters remained within normal ranges, and excessive fluid administration was avoided. Re-expansion pulmonary edema (RPE) is one of the most severe complications of MICS with right mini-thoracotomy [5]. Although chest radiography of the RPE demonstrated unilateral pulmonary edema, this case showed bilateral infiltration, which was unlikely to be diagnosed as RPE. 

Moreover, transfusion-related acute lung injury (TRALI) is a rare differential diagnosis that can be considered a cause of acute respiratory failure following cardiac surgery. TRALI is defined as the acute onset of hypoxia and bilateral pulmonary infiltrates after allogeneic blood transfusion [6]. However, in this case, referring to the new criteria for TRALI [7], the features of acute lung injury emerged more than six hours after transfusion, which did not correspond with the diagnosis of TRALI.

After excluding other differential diagnoses, we diagnosed ARDS based on established diagnostic criteria [8]. Longer periods of CPB, the use of hypothermic circulatory arrest, patients undergoing aortic surgery, and left ventricular assist device placement are known risk factors of ARDS during the perioperative period of cardiac surgery [9]. Although our patient did not have any such risk factors, cardiac surgery is a known risk factor for ARDS [9]. The reported incidence of ARDS after cardiac surgery varies widely, ranging from 0.4 to 8.1% [10]. Therefore, the possibility of ARDS must be considered when encountering respiratory failure following cardiac surgery.

Pharmacotherapy options for ARDS, such as methylprednisolone or Sivelestat, remain controversial. While the use of inhaled NO can be effective on a case-by-case basis, its drawbacks, including the potential of inducing free radical injury to the lungs, should be considered [9,10]. In contrast, early administration of mechanical ventilation is recommended when ARDS is suspected [11]. We achieved an improvement in the respiratory status after re-intubation within a short period. Although maintaining oxygenation with normal mechanical ventilation is difficult, the early introduction of extracorporeal membrane oxygenation is recommended [11]. 

## Conclusions

We discussed a case of ARDS after MICS treatment. ARDS occurring after cardiac surgery is a rare but lethal condition. We have to strongly consider the possibility of ARDS following cardiac surgery, and early intervention is crucial. Ventilator management strategies considered effective for ARDS should be implemented promptly to achieve favorable outcomes in these patients.
